# Upper limb home-based robotic rehabilitation in chronic stroke patients: A pilot study

**DOI:** 10.3389/fnbot.2023.1130770

**Published:** 2023-03-16

**Authors:** Federica Bressi, Benedetta Campagnola, Laura Cricenti, Fabio Santacaterina, Sandra Miccinilli, Giovanni Di Pino, Francesca Fiori, Marco D'Alonzo, Vincenzo Di Lazzaro, Lorenzo Ricci, Fioravante Capone, Alessandra Pacilli, Silvia Sterzi, Marco Bravi

**Affiliations:** ^1^Physical Medicine and Rehabilitation Unit, Campus Bio-Medico University of Rome, Rome, Italy; ^2^Research Unit of Neurology, Neurophysiology and Neurobiology and Biomedical Robotics and Biomicrosystems, Campus Bio-Medico University of Rome, Rome, Italy; ^3^Unit of Neurology, Neurophysiology and Neurobiology, Department of Medicine, Campus Bio-Medico University of Rome, Rome, Italy; ^4^Heaxel, Milan, Italy

**Keywords:** home-based, stroke, robotic device, upper limb, rehabilitation, technologies

## Abstract

**Introduction:**

Robotic therapy allow to propose sessions of controlled and identical exercises, customizing settings, and characteristics on the individual patient. The effectiveness of robotic assisted therapy is still under study and the use of robots in clinical practice is still limited. Moreover, the possibility of treatment at home allows to reduce the economic costs and time to be borne by the patient and the caregiver and is a valid tool during periods of pandemic such as covid. The aim of this study is to assess whether a robotic home-based treatment rehabilitation using the iCONE robotic device has effects on a stroke population, despite the chronic condition of patients involved and the absence of a therapist next to the patient while performing the exercises.

**Materials and methods:**

All patients underwent an initial (T0) and final (T1) assessment with the iCONE robotic device and clinical scales. After T0 evaluation, the robot was delivered to the patient's home for 10 days of at-home treatment (5 days a week for 2 weeks).

**Results:**

Comparison between T0 and T1 evaluations revealed some significant improvements in robot-evaluated indices such as Independence and Size for the Circle Drawing exercise and Movement Duration for Point-to-Point exercise, but also in the MAS of the elbow. From the analysis of the acceptability questionnaire, a general appreciation of the robot emerged: patients spontaneously asked for the addition of further sessions and to continue therapy.

**Discussion:**

Telerehabilitation of patients suffering from a chronic stroke is an area that is still little explored. From our experience, this is one of the first studies to carry out a telerehabilitation with these characteristics. The use of robots can become a method to reduce the rehabilitation health costs, to ensure continuity of care, and to arrive in more distant places or where the availability of resources is limited.

**Conclusion:**

From the data obtained, this rehabilitation seems to be promising for this population. Moreover, promoting the recovery of the upper limb, iCONE can improve patient's quality of life. It would be interesting to conduct RCT studies to compare a conventional treatment in structure with a robotic telematics treatment.

## Introduction

Stroke is the second leading cause of death and a major cause of disability worldwide. Currently it is estimated that every year about 33 million people suffer a stroke, but this incidence is expected to increase due to the progressive aging of the population (Wang et al., 2016; Soriano et al., 2017; Katan and Luft, 2018). Typically, 1 year after stroke 65% of these patients remain severely impaired and the degree of disability is correlated with the severity of stroke (McConnell et al., 2017); this translates into an increase in assistance for carrying out the activities of daily living (ADL). Motor impairment is the most common consequence of stroke, which can be regarded as loss or limitation of function in muscle control or movement in an arm and a leg on one side of the body (Pollock et al., 2014). Upper limb weakness is a common condition, affecting about the 85% of survivors. Therefore, one of the main aims of rehabilitation is to improve upper limb functions.

Evidence in the literature underlined how motor training can positively influence the recovery by enhancing brain plasticity after stroke, especially, when a multisensory rehabilitation is proposed (Poli et al., 2013) and when repetitive and task-oriented exercises, with a high number of repetitions, are delivered. This type of rehabilitation requires great commitment for both patients and physiotherapists, resulting in high costs for the health care system (Jenkins and Merzenich, 1987; Masiero and Carraro, 2008; Poli et al., 2013). Robotic devices can help overcome these obstacles indeed, the use of these devices has been proposed since the 90s to help therapists increase the intensity of sessions, provide multisensory stimulation, and reduce costs (Poli et al., 2013; McConnell et al., 2017). Furthermore, robotic devices allow to propose sessions of controlled and identical exercises, tailored on the characteristics of the individual patient (Li et al., 2022). Moreover, through the use of screens and visual feedback, robotic devices provide sensory input, encouraging learning thanks to the increased involvement given by the interactivity of the technological device (Maciejasz et al., 2014).

The effectiveness of robotic assisted therapy is still under study and the use of robots in clinical practice is still limited. One of the reasons may be related to the logistics of using these devices. In fact, the patients for whom the use of the robot is indicated are generally severely disabled, requiring the assistance of a caregiver to get to visits and therapeutic sessions (Li et al., 2022). The solution to this problem could be the use of robotic devices for home rehabilitation: most of the recent robotic rehabilitation devices are designed and built to be transported to the patient's home, so that the patient can perform the exercises several times a day. Home treatments can also be provided using devices such as smartphones and tablets or through the use of webcams. A recent review reported that in motor recovery after stroke, telerehabilitation appears to have similar results to clinical rehabilitation. According to this review, both for sub-acute and chronic patients, technological rehabilitation programs should be integrated into conventional therapy (Maciejasz et al., 2014). These results are also supported by a recent Cochrane review by Laver et al., in which 22 Randomized Controlled Trials were analyzed, for a total of 1,937 patients. This review shows that there is no difference in daily life activities between people who at discharge have received telerehabilitation and those who have received regular care (Laver et al., 2020). However, the few studies in the literature show conflicting results, so the aim of this pilot study is to evaluate the effectiveness of a robotic home treatment rehabilitation on patients suffering from chronic stroke. This study also allows to analyze another important aspect: the effectiveness of robotic therapy in the absence of a physiotherapist alongside the patient. In addition, an acceptability questionnaire was administered to assess patients and caregivers' satisfaction of the robot and whether robotic therapy increases the workload of the caregiver. In fact, although the commitment related to the transport of the patient to the rehabilitation site is eliminated, the involvement of the caregiver cannot be eliminated, but the workload can be reduced by ensuring the maximum flexibility of the therapy.

## Materials and methods

### Study design

The present study is a monocentric pilot study on the use of the iCONE robotic device (Heaxel srl, Milan, Italy) for home rehabilitation of patients with chronic stroke outcome approved by the ethics committee of the Fondazione Policlinico Universitario Campus Bio-Medico (protocol number 29/19).

This study aims to assess whether a robotic home-based treatment rehabilitation delivered by the use of the iCONE robotic device for two consecutive weeks has effects on this population, despite the chronic condition of patients involved and the absence of a therapist next to the patient while performing the exercises. Two evaluations were provided in order to assess the effectiveness of treatment: before the start of treatment and at the end of treatment. The evaluations included the administration of clinical scales by medical staff and physiotherapists and the execution of exercises provided by the robot. The exercises administered for the evaluation with the robot were the same for all patients and for both evaluations.

### Participants

The study involved patients recruited in the period between March 2021 and April 2022 by the Complex Operative Unit (COU) of Physical Medicine and Rehabilitation, the COU of Neurology, and the Research Unit of Neurology, Neurophysiology and Neurobiology and Biomedical Robotics and Biomicrosystems of the Campus Bio-Medico University Hospital Foundation of Rome. Enrolled patients underwent clinical examination before the initial assessment.

Inclusion criteria for this study included patients aged between 18 and 80 years, with chronic stroke outcome (stroke onset at least 6 months before treatment) and residual upper limb deficiency evaluated with an Upper Limb Fugl-Meyer Assessment ≥3. Patients also had to be able to understand the indications given by the therapist and the ability to sign the informed consent. Moreover, no patients who had criteria contrary to the conditions required by the use of the robot were recruited. These criteria were: a positive history of epilepsy, presence of severe cognitive deficits and/or psychiatric disorders, severe flaccidity of the upper limb, lack of balance of the trunk in sitting position, and orthopedic pathologies of the upper limb that made it difficult to use the robot.

### General intervention description

After recruitment, patients underwent an initial assessment (T0) through a series of clinical scales to assess the degree of cognitive and functional disability of the patient, autonomy in ADL, motor skills, and the degree of spasticity. In particular, the NIHSS, the Barthel Index, the Modified Ranking Scale, the Fugl-Meyer Assessment for the upper limb, and the Modified Ashworth Scale were administered. The detailed description of the scales is given below. Patients were then subjected to a motor and performance assessment using the robotic device iCONE, in order to evaluate parameters such as coordination and muscle synergy, precision, fluidity of movement, strength.

**Figure 1:**
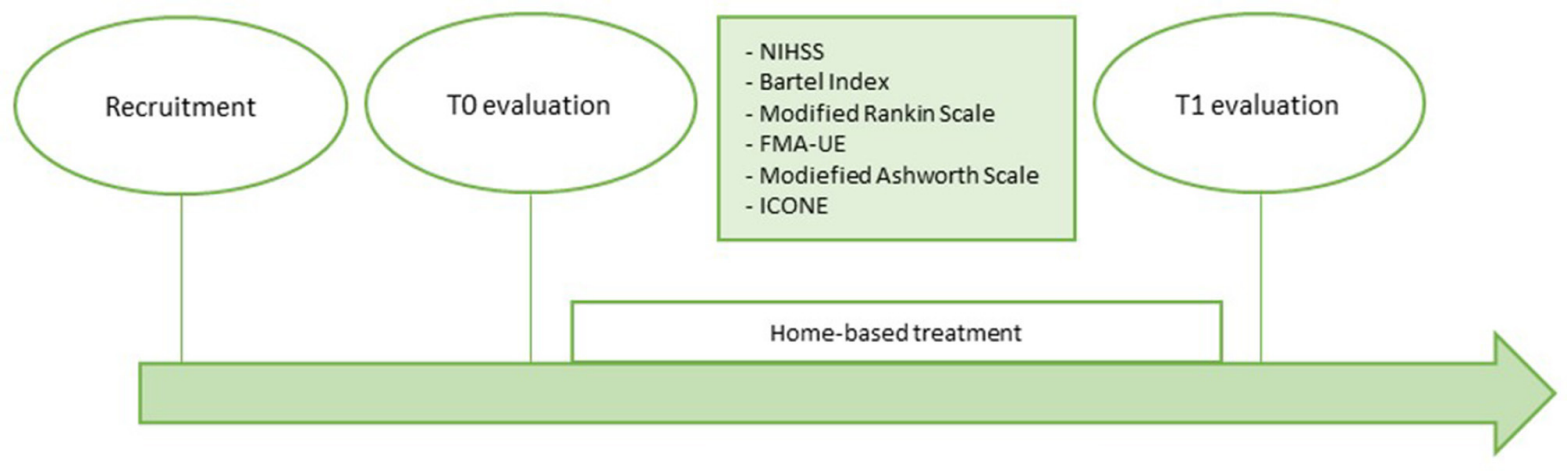
Timeline.

The home-rehabilitation included 10 sessions of exercises performed with the iCONE device with the supervision of a caregiver previously trained by the engineering team on robot management. At the end of the 2 weeks of home treatment a second evaluation (T1) was repeated in the same way as the T0. Figure 1 shows the timeline of the study.

The exercises proposed in the evaluation do not coincide with those proposed in the exercises at home. This prevents the results obtained in the T1 assessment from being affected by the exercise training component.

### The iCONE robotic device

**Figure 2:**
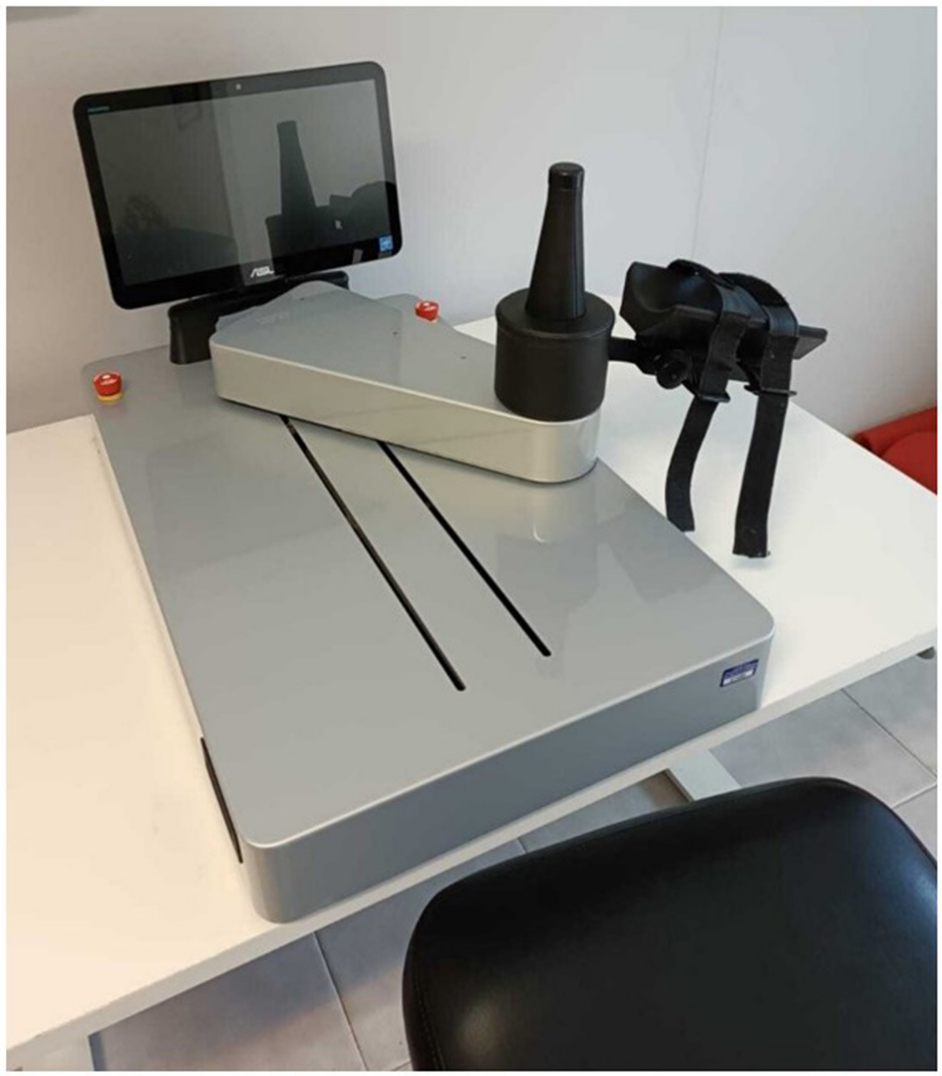
The iCONE robotic device.

iCONE (Heaxel, Milan, Italy) is a medical device for robot-assisted neurorehabilitation of the upper limb (Figure 2). It consists of a metal structure connected to a handle that allows the movement on the transverse plane of the upper limb and a monitor that shows the exercise to be carried out. The handle is interchangeable to adapt to the patient's grip. It requires the movement both of shoulder and elbow, while the hand is not involved and is anchored to the handle. The robot comes with a table adjustable in height to adapt to the most comfortable seat of the patient.

At the moment of taking charge of the patient, the robot provides to create a user card in which the date of the event, the type, and the injured side can be recorded. iCONE has the authorization for use in both healthcare facilities and in non-hospital environment. For this reason, it can be used at the patient's home favoring the possibility of telerehabilitation.

The robot allows the administration of protocols based on the intensive repetition of therapeutic exercises and integrates a computer that indicate on the display the specific points that must be reached in the movement, requiring shoulder and elbow coordination to perform tasks of reaching. The use of the screen also provides visual feedback for the patient.

The iCONE robotic device can perform two types of session: the evaluative session and the therapy session. The evaluative session involves performing six standard exercises and provides a complete report extrapolating quantitative indicators from the planned exercises. These indicators are shown both numerically and graphically and when a new evaluation is made, the data of the previous evaluations are reported alongside the new ones, facilitating comparison. For each indicator, or index, a description is given to define what it refers to. Moreover, the expected trend is reported.

The six exercises proposed for the evaluation and the respective indices are described below:

- **Circle drawings**: it requires to draw a total of twenty circles divided into four series of five repetitions for different directions: the circles must be drawn clockwise and counterclockwise, starting from the left and then from the right, so that there will be five circles from left to right passing from the top and five circles passing from the bottom, and five circles from right to left from the top and five from the bottom. The therapist gives the start and the stop for each circle. The robot is in a transparent mode, which means that it provides neither assistance nor resistance to the patient during the execution of the task. It provides two indexes: independence and size.

° *Independence*: it is the ratio of the minor axis to the main axis of the ellipse that adapts to the circles drawn by the patient. An increase is expected as therapy progresses (a perfect circle would have a ratio of 1.00). Higher values indicate better coordination and synergistic control of the elbow and shoulder.
° *Size:* it is the total area of the ellipse that best suits the circles drawn by the patient. An increase is expected as the therapy proceeds. Indicates improvements in the Range of Motion (ROM) of the paretic limb.

- **Point-to-point**: it consists in the classic round of the clock in which the patient must reach the eight targets arranged along the perimeter of a circle starting each time from the center. The exercise includes five clock turns. During the exercise the robot is in transparent mode. It provides seven indexes: init time, mean speed, movement duration, path error, reach error, smoothness, and peak speed.

° *Init time:* it indicates the time needed to start the movement independently. It is expected to decrease as therapy progresses. It is an indicator of the ability of planning movement.
° *Mean speed:* Average speed of the end-effector in the execution of the point-point movements. The average speed is expected to increase as therapy progresses.
° *Movement duration:* Average time needed to perform a point-to-point movement. It is a measure of temporal efficiency. It decreases as therapy progresses.
° Path error: Average distance of each point in the patient's trajectory from the theoretical path. It measures the accuracy of the entire reaching movement. Decreases as therapy progresses (ideally zero).
° *Reach error:* Indicates how close the patient is to the target, on average. It is a precision measurement. Lower result indicates better performance. A radar graph shows the value of the indicator along each direction of movement.
° *Smoothness (speed shape):* Ratio between average and maximum speed during point-to-point movements. It is an indicator of fluidity and ease of execution of the movement: the higher the value, the easier it is for the patient to complete the movement. It increases with the progress of therapy.
° *Peak speed:* Peak speed value of the patient. Indicates ease of movement. Increases as therapy progresses.

- **Playback static:** requires the patient to hold the handle of the robot in the center, while the robot moves toward the eight targets. This exercise provides one index: hold deviation.

° *Hold deviation:* It represents the average deviation from the center during the Playback Static exercise. A patient with a flaccid limb may show a star-shaped movement. The indicator tends to shrink as therapy proceeds.

- **Round dynamic:** it is the opposite of the previous exercise. Requires that the patient brings the robot's end-effector to the eight targets, while the robot applies a resistance to the center. It provides the index displacement.

° *Displacement:* Average distance covered against resistance from the central target during Round Dynamic exercise. The value increases with the progress of therapy.

- **Shoulder horizontal abduction**: the straps on the forearm are loosened and only the hand remains in contact to the end-effector. The patient is positioned with the elbow extended and the shoulder at 90°. The handle of the robot freezes in the middle of the screen and the patient is asked for a 5 s shoulder abduction. The exercise is repeated five times. It provides one index: shoulder horizontal abduction.

° *Shoulder horizontal abduction:* Maximum variation of force exerted by the patient during the 5 s of the five repetitions, while trying to remove the end-effector from its sagittal plane. It increases in the course of therapy.

- **Shoulder horizontal adduction**: the conditions are the same as the previous exercise, but the movement required is an abduction. It provides one index: shoulder horizontal adduction.

° *Shoulder horizontal adduction:* Maximum variation of force exerted by the patient during the 5 s of the five repetitions, while trying to bring the end-effector to its sagittal plane. Increases in the course of therapy.

**Figure 3:**
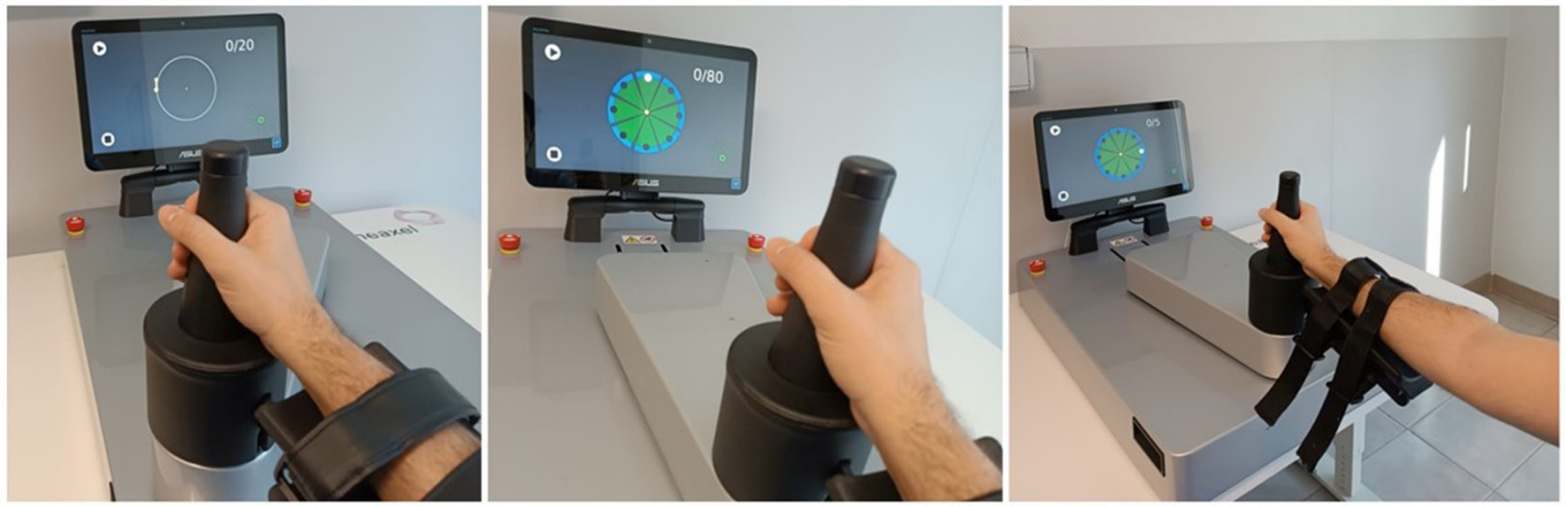
Evaluation exercises: circle drawing, point to point e horizontal abduction/adduction.

**Figure 4:**
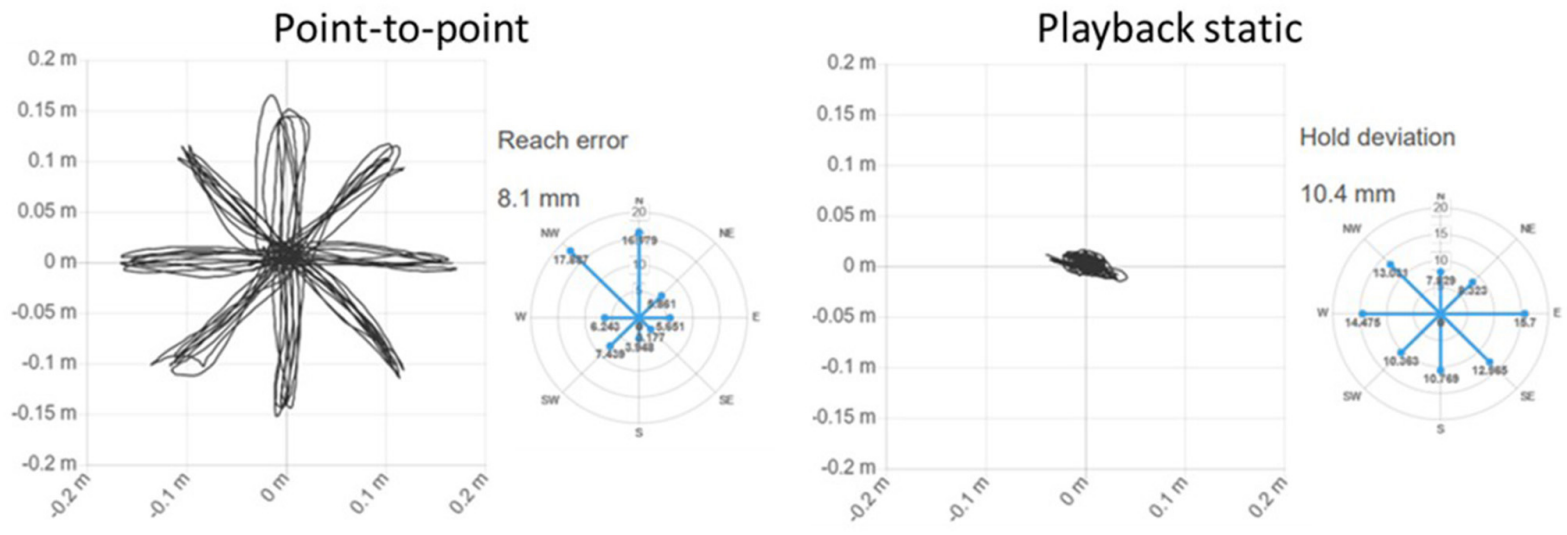
Example of trajectories and indices of two exercises.

Figure 3 shows the screen shown to the patient in the exercises Circle drawing, Point-to-point e Horizontal Abduction/Adduction, while Figure 4 shows the trajectories and two examples of indices for the exercises proposed in the evaluation.

While exercises for evaluation are standard, for therapy several parameters can be set. In particular, the width and stiffness of the haptic tunnel, the level of assistance and resistance, the number of repetitions for exercise, the accuracy of the target, the scenery can be changed. In addition, it can be customized the waiting time for the start of the movement before the robot activates to help the patient and the maximum assigned time to complete the movement before the robot passes to the next target. There are three modes of interaction to adapt to each degree of disability of the patient: assistive, resistive, or adaptive. The assistive mode requires the robot to help the patient perform tasks and allows a different degree of assistance to be set according to the patient's conditions. The resistive mode allows to set up a resistance training for patients who have achieved a good degree of movement control and who need muscle reinforcement. The adaptive mode requires the robot to help the patient only when the movement can't be completed. This study has adopted the assistive modality in order to adapt to all the levels of impairment.

An important feature of this device is the ability to follow the patient remotely by the cloud. In fact, therapy progress and session execution data are constantly updated and stored in the cloud, so that the therapist can monitor and change the training sessions at any time by remotely accessing the device delivered to the patient thanks to the wi-fi connection. For this purpose, in case the patient was not provided with wi-fi, a router was provided with the device.

### Intervention

All patients included in the study carried out an initial (T0) and final (T1) assessment administered at the CESA (Health Center of the Elderly) of Campus Bio-Medico University Polyclinic Foundation of Rome.

The two evaluations included the administration of the following clinical scales: National Institute of Health Stroke Scale (NIHSS), Modified Rankin Scale (MRS), and Barthel Index (BI) submitted by neurologists; Fugl-Meyer Assessment for upper extremity (FMA-UE) and Modified Ashworth Scale (MAS) administered by physiotherapists.

In this study, NIHSS, MRS, and BI were administered with the aim to frame the degree of disability and severity of the included patients.

The NIHSS is used to measure stroke severity. It is composed of 15 items that investigate level of consciousness, eye movements, integrity of visual fields, facial movements, arm and leg muscle strength, sensation, coordination, language, speech, and neglect. For each domain, a score ranging from 0 to 2, 0 to 3, or 0 to 4 can be recorded. The total score ranging from 0 to 42 is calculated as the sum of individual item scores. The higher the score, the more severe the stroke (Kwah and Diong, 2014).

The MRS was developed to measure the disability or dependence in the daily activities of people with stroke outcomes or other neurological disorders (Haggag and Hodgson, 2022). It is composed of a single item ranging from 0 to 6, where 0 means no symptoms, 1 no significant disability, 2 slight disability, 3 moderate disability, 4 moderately severe disability, 5 severe disability, and 6 refer to death (Broderick et al., 2017).

The BI measures 10 basic aspects of activity related to self-care and mobility. It investigates 10 items (feeding, grooming, bowel and bladder management, toilet use, dressing, bathing, transfer, mobility, and stairs) with a score ranging from 0 to 10 or 0 to 15. The normal score for this scale is 100 points and lower scores indicate greater dependency (Kasner, 2006).

The FMA-UE has different domains: it evaluates the motor aspects, the sensitivity, the passive ROM and the pain. The Motor function has a maximum score of 66 points. The evaluation investigates voluntary movement, speed, coordination, and reflex activity. For each item a score ranging from 0 to 2 can be assigned depending on the ability to perform and complete the task: 0 = cannot be performed, 1 = partially performed, 2 = performed completely. The total score allows to classify the motor impairment as severe (< 32 points), moderate (between 32 and 47), or mild (>47 points) (Barbosa et al., 2019; Rech et al., 2020). Sensitivity is evaluated both as light touch and proprioception and it has a maximum score of 12. Passive ROM and joint pain are evaluated for all districts. The passive ROM has a maximum score of 24, the pain has a maximum score of 12. As for the motor function, for these domains the scores for each item ranges from 0 to 2.

MAS is used to evaluate passive movement resistance. This scale allows to obtain an indirect assessment of spasticity. The score ranges from 0 (no tonus increase) to 4 (stiffness). Patients are evaluated in a lying position, and they are asked to remain relaxed during the test (Maciejasz et al., 2014; Rech et al., 2020).

In addition, patients also performed an evaluation session with the robot consisting of a series of six exercises as described previously. The exercises provided by the robot for evaluation are standardized to always be the same for all patients. At the end of the evaluation, the robot provides a report with numerical indexes comparable between the two evaluation times and graphs with the trajectories followed by the patient during the exercises.

At the final evaluation, an acceptability questionnaire was administered consisting of a question for the patient about the difficulty of using the robot and the possibility to integrate it in the daily activities, and one for the caregiver to assess how much the required workload in patient care has increased. It was also required to quantify this feature giving a score ranging from 0 to 10. The acceptability questionnaire is available in the Italian version and in an English translation in the Supplementary material.

After initial evaluation, the robot was delivered to the patient's home. The rehabilitation protocol provided 10 days of at-home treatment (5 days a week for 2 weeks).

The robot allows to plan therapies with customizable sessions. Each session consisted in point to point reaching exercises for a total of 1,024 reaching movements. For each session we adapted the number of consecutive repetitions, depending on the patient's need to make breaks with greater or lesser frequency.

For this protocol, a typical session was as follows: four exercises of 16 repetitions and six exercises of 160 repetitions according to the following scheme: 16-160-160-16-160-160-16-160-160-16.

For patients with increased motor impairment, it was necessary to further divide the session, to allow the patient to intersperse the exercises with more frequent breaks. For these patients each of the exercises of 160 repetitions was divided into five exercises of 32. This distinction was based on the difficulties reported by the individual patient. In the first days of therapy the physiotherapist assessed the need to divide the session.

The total number of repetitions for each session to be performed throughout the day was fixed at 1,024 movements for each patient.

Despite the customization of the rehabilitation, some patients had difficulty to complete all the required tasks, while others exceeded in the sessions for which it was necessary to add some more to complete the 10 days of therapy.

During the home treatment the patients were followed exclusively by a caregiver (usually a family member of the patient) designated by the therapist at the initial evaluation or by the engineer at the time of delivery of the robot at home. The caregiver did not have an active role in the therapy, but his presence is required for the use of the robot and his purpose is to intervene in case of need or adverse events.

It is not possible to establish the overall commitment of the treatment as this varies greatly from patient to patient: for some patients it took an hour a day to complete all the exercises, while others took up to 3 h.

Physiotherapist had the ability to follow the patient remotely via cloud, so that it was possible to add a new session wherever the patient exceeded daily therapy or reduce the number of tasks per exercises if the patient required more breaks. When many incomplete exercises appeared or abnormalities were found, the therapist contacted the patient by phone to make sure the therapy was adequate.

### Data analysis

Normality assumptions were tested by means of the Shapiro-Wilk test (*p* > 0.05) and data were then analyzed with the appropriate statistical test. Technical failure caused a lack of data in some conditions, in fact, in some cases the robot has not recorded the full values of the indices, especially when patients made incomplete movements or were not able to complete the assessment exercise. Thus, data were analyzed with generalized linear mixed models (GLMM) and, when necessary, scaled with a (min–max) + 1 normalization to obtain positive numbers > 1. This has made it possible to carry out a more complete analysis, since unlike other methods, the GLMM allow to analyze the data albeit partial, avoiding data loss. Moreover, for the same reason also allows to use data of the drop out patient. We chose the most appropriate family and link function as the model with the lowest Akaike information criterion (AIC).

All the reported results were corrected, when appropriate, with Holm correction for multiple comparisons. Statistical significance was set at *p*-values < 0.05.

## Results

**Figure 5:**
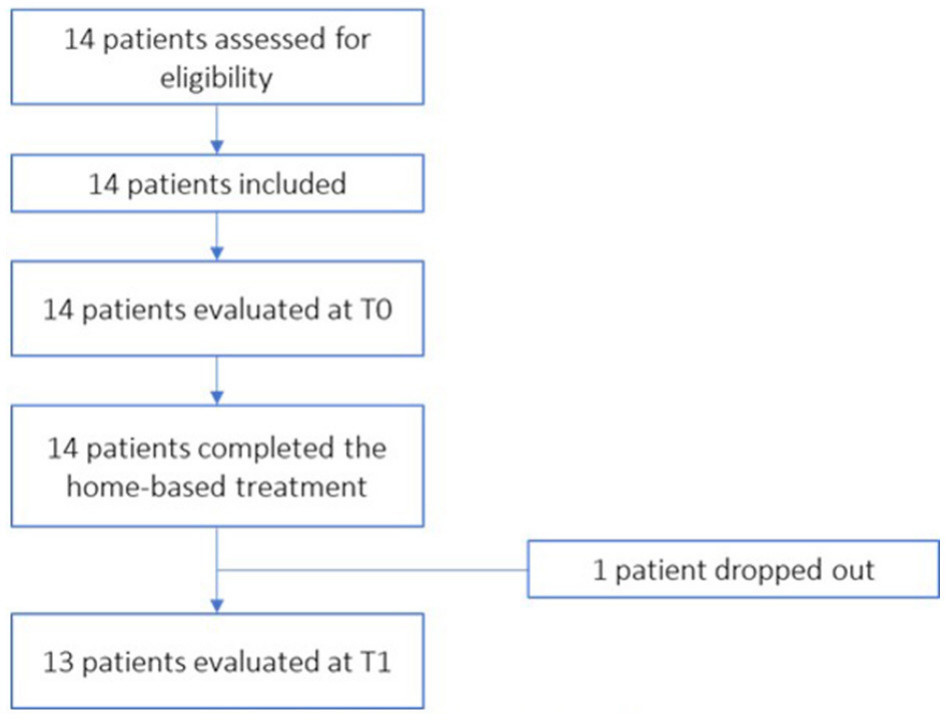
Participants flow diagram.

All the patients recruited were evaluated and considered suitable for treatment as regards the eligibility criteria. A total of 14 patients were included in the treatment. Figure 5 shows the flow diagram of patient recruitment.

The patients recruited were all adults, with an average age of 59.29 years (between 32 and 79 years) suffering from chronic, ischemic or hemorrhagic stroke (respectively, 11 vs. 3 cases). They had residual upper limb disabilities due to a first ever stroke and the minimum time away from the cerebrovascular event was at least 6 months from the start of the study. They were predominantly women (8F/6M) and the affected side was equally distinct among the participants: seven patients had a right hemisphere injury and seven had a left hemisphere injury.

Data regarding demographic information of the included patients are reported in Table 1.

**Table 1:** Characteristics of the patients.

**Participants (no. 14)**	**Age**	**Sex**	**Type of stroke**	**Affected limb**	**Time from stroke (months)**	**NIHSS**	**BI**	**MRS**
Patient 1	73	M	I	L	177.97	2	75	3
Patient 2	55	F	I	L	188.39	4	75	4
Patient 3	64	M	H	R	32.49	2	75	3
Patient 4	52	M	H	L	113.97	4	80	2
Patient 5^*^	59	M	I	R	28.98	8	85	2
Patient 6	32	F	I	R	192.00	3	95	1
Patient 7	69	M	I	R	6.74	5	61	3
Patient 8	78	F	I	L	8.25	3	90	1
Patient 9	46	F	I	R	22.21	3	98	1
Patient 10	63	F	I	R	6.12	7	85	3
Patient 11	58	F	I	L	701.04	3	95	1
Patient 12	79	F	I	L	14.06	10	30	4
Patient 13	53	M	H	R	343.46	6	89	3
Patient 14	49	F	I	L	37.72	2	90	2
Mean ± sd	59.29 ± 13.0	6M/8F	11I/3E	7L/7R	133.8 ± 192.1	4.43 ± 2.47	80.21 ± 17.63	2.36 ± 1.08

M, male; F, female; I, ischemic; H, hemorrhagic; NIHSS, National Institutes of Health Stroke Scale; BI, Barthel Index; MRS, Modified Ranking Scale.

^*^Patient 5 dropped out after treatment.

During the study a voluntary drop-out was recorded related to problems of the patient's family (patient 5): it was not possible for family members to bring the patient to the T1 evaluation. Then, of the 14 patients recruited, 13 completed all evaluations.

Data obtained from the NHISS, BI, and MRS were reported to describe the clinical status of the sample analyzed. Table 1 shows the values recorded for each patient and the average and standard deviation for the total of the patients.

Table 2 show the results obtained in the Fugl-Meyer Assessment divided for the different domain and the Ashworth scale evaluation for shoulder, elbow, and wrist districts. Data were analyzed with GLMM method. Average and standard deviation are reported for each data for both T0 and T1. Moreover, statistical significance is reported for the comparison between T0 and T1.

**Table 2:** FMA-UE and MAS comparison between T0 and T1.

	**T0**	**T1**	***p*-value**
	**(mean ± sd)**	**(mean ± sd)**	
FMA motor function	33.21 ± 14.87	35.69 ± 15.92	0.882
FMA sensibility	10.36 ± 2.71	10.92 ± 2.25	0.952
FMA passive ROM	18.71 ± 3.07	18.92 ± 3.09	0.985
FMA pain	21.21 ± 2.78	21.69 ± 2.69	0.296
MAS shoulder	1.07 ± 0.73	0.92 ± 0.95	0.113
MAS elbow	1.86 ± 0.95	1.46 ± 1.2	0.017^*^
MAS wrist	1.36 ± 0.93	1.46 ± 1.13	0.955

FMA, Fugl-Meyer Assessment; MAS, Modified Ashworth Scale.

The ^*^ symbol indicates values that reach statistical significance.

The comparison of the results obtained in the FMA-UE between T0 and T1 highlights a substantial stability at T1 for all the domains analyzed, with a slight but not significant improvement in motor function.

The MAS for the elbow district reported a statistically significant result in the comparison between T0 and T1.

In the analysis of robot indices data were analyzed with GLMM and, when necessary, scaled with a (min–max) + 1 normalization to obtain positive numbers > 1. We chose the most appropriate family and link function as the model with the lowest AIC. Table 3 summarize mean, standard deviation, and *p*-values related to the comparison between the two times T0 and T1 for all the variables.

**Table 3:** Comparison between T0 and T1 for iCONE indices.

**Index**	**T0**	**T1**	***p*-value**
	**(mean ± sd)**	**(mean ± sd)**	
Independence	0.59 ± 0.39	0.76 ± 0.25	0.040^*^
Size	0.04 ± 0.03	0.05 ± 0.02	0.019^*^
Init time	0.14 ± 0.23	0.12 ± 0.28	0.916
Mean speed	0.07 ± 0.03	0.08 ± 0.05	0.073
Movement duration	3.53 ± 1.46	2.66 ± 1.52	0.002^**^
Path error	13.02 ± 5.17	11.12 ± 4.49	0.218
Reach error	20.46 ± 14.81	18.72 ± 17.59	0.993
Smoothness	0.54 ± 0.09	0.57 ± 0.12	0.514
Peak speed	0.12 ± 0.04	0.14 ± 0.07	0.168
Hold deviation	26.78 ± 21.1	24.97 ± 16.89	0.448
Displacement	58.65 ± 22.71	64.07 ± 15.59	0.068
Shoulder horizontal abduction	20.84 ± 9.36	23.24 ± 5.89	0.273
Shoulder horizontal adduction	21.72 ± 12.12	23.9 ± 6.5	0.444

The ^*^ and ^**^ symbols indicate values that reach statistical significance. A single asterisk for p-values ≤ 0.05; two asterisks for p-values ≤ 0.01.

**Figure 6:**
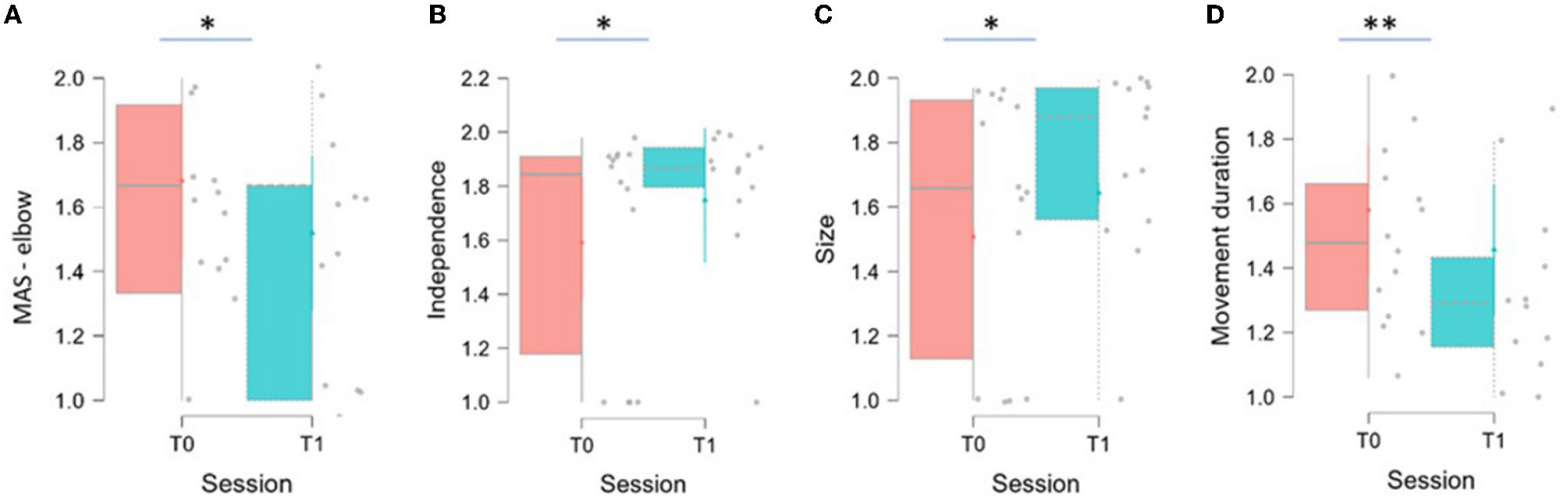
Graphs related to significant data.

As reported in Table 3, statistically significant results have been recorded for three indices: Independence significantly increased between T0 and T1 (*p* = 0.040), Size significantly increased between T0 and T1 (*p* = 0.019), and Movement Duration significantly decreased between T0 and T1 (*p* = 0.002). The distribution of the variables at the two evaluations are shown in Figure 6.

Figure 6 shows box and whisker plot of MAS for the elbow district, Independence, Size, and Movement Duration represented in A, B, C, D, respectively. The thick horizontal gray line within the boxplot represents the median value. Asterisks indicate significant differences (^*^p < 0.05, ^**^p < 0.01).

Two variables have achieved interesting results although not statistically significant: Mean Speed (*p* = 0.073) and Displacement (*p* = 0.068). Both variables are increased between T0 and T1.

From the results obtained in the acceptability questionnaire, all the patient (100%) stated that the treatment proposed at home was compatible with daily life activities. The average score given to the liking of the robot was 9.23 points on a scale from 0 to 10. Ten caregivers (76.92%) reported that their care load did not increase during the home-based treatment, with a score of 0 on a scale from 0 to 10, while three of them (23.08%) reported that their workload increased with an average score of 7.3.

No adverse events were recorded during the evaluations or the home-based treatment.

## Discussion

The purpose of the present study was to assess whether a rehabilitation treatment delivered at home using a robotic device could have a positive effect on patients with chronic stroke outcome, even when the therapist is not next to the patient while performing the exercises. The results obtained from our study seem to support the hypothesis of the usefulness of this type of rehabilitation.

The significant data obtained in the Independence, Size, and Movement Duration relate to the synergy of movement, the ROM, and the duration of movement, indicating an improvement of the patient in performing wider movements, with better control, greater fluidity, and shorter time. This could result in improved functionality of the paretic limb, which has been reported by many of the patients, even if it has not been found in the clinical scales administered. This result is in line with the results obtained in the evaluation with MAS. In fact, the significant decrease in tone at the elbow of the examined limb may have contributed to the greater fluidity of the movement, indicating that this device is suitable for use in these patients, avoiding causing an increase in spasticity.

The two indices Mean Speed and Displacement reached very interesting even if not significant values. These variables refer, respectively, to the speed and to the distance covered against resistance in the execution of the reaching movement. The increase of these two parameters could be a next step in the improvement of the movement management, as it provides for a good control by the patient even during the application of a force.

Moreover, the results obtained in the robotic evaluation cannot be linked to the learning in the use of the device by the patient, in fact the exercises proposed in the treatment are different to those proposed in the evaluation. Consequently, it was not possible to find a clear response and support to our results, although these seem favorable not only for the outcomes obtained by patients, but also for the management of therapy.

As reported by Chen et al. in their systematic review, the results obtained from home treatment with these devices are very discordant. Several studies reported that patients treated with robotic devices can achieve improvements comparable to those treated with conventional therapies. Alongside these studies, however, there are numerous studies that report no statistically significant results in the same comparison (Chen et al., 2019).

Although the robotic indices reported several significant data, the scales of evaluation of the functionality did not show major changes, in particular the FMA-UE. This could be explained by a higher sensitivity of indices compared to clinical scales that require greater variation to record changes in scores.

For the duration of treatment, as already mentioned, it was not possible to make a direct comparison with studies involving the use of the same robot, so it was compared with robots having similar structure and the same degrees of freedom, for example the MIT-Manus device (Massachusetts Institute of Technology, Cambridge, MA, USA). The analyzed studies took into consideration the treatment in structure and not at home. This analysis showed that treatment times vary from 4 to 6 weeks for this type of devices, with a frequency of 5 days a week (Volpe et al., 2000, 2008; Ang et al., 2014; Sale et al., 2014). Therefore, it would be interesting to increase the treatment period to assess whether this produces an improvement in the results or an abandonment of the therapy.

According to the questionnaires administered at T1, patients were satisfied with robotic therapy, showing that the device was found to be well-tolerated by the patients. Therefore, caregivers reported that their workload is not increased during the home-based treatment, not affecting the organization of daily activities.

The purpose of post-stroke rehabilitation is to promote the recovery of lost functions to allow the patient to achieve independence and reintegration into social life. To date there are no studies in the literature carried out with this robotic device and there are still few studies about the use of robots at the patient's home. Even if they are not yet widespread, robotic systems offering home rehabilitation for patients with neurological diseases are becoming increasingly known and accepted (Guillén-Climent et al., 2021). Robotic devices for the therapy of the upper limb enabled to operate at the patient's home present several advantages. In fact, patients can perform the established therapy at any time of the day, without the need to reach a rehabilitation facility with consequent less impact on the caregiver's load. Moreover, the costs relative to the attainment of the structure and the times of employment demanded to the caregiver are significantly reduced.

An important factor in favor of home robotic therapy is the possibility of providing high-dose rehabilitation therapy even to those patients with chronic outcomes, for whom the journey within hospital facilities has ended. This type of rehabilitation at home could therefore represent a valid alternative to the management of chronic diseases, guaranteeing this category of patients an adequate treatment to maintain longer the autonomy achieved in the acute phase. Moreover, the interactivity of telerehabilitation and the possibility of modulating the intensity of treatment are useful to adapt the therapy to the progress of the patient (Cramer et al., 2019) and possibility to vary the proposed games and actions enable these devices to always being stimulating (Nijenhuis et al., 2015). The proposal of exercises in the form of play, can increase the patient's involvement and consequently his adherence to therapy (Popović et al., 2014; Rodríguez-De-Pablo et al., 2016).

Home robotic rehabilitation also has disadvantages: using robotic devices at home concerns the need for ample space for placement. This can be a problem for subjects living in apartments with little spaces. In addition, in some cases robots produce forces that can affect the safety of the treatment (Chen et al., 2019). For this reason, the iCONE provides the presence of a caregiver during treatment trained to stop the robot in the event of problems or risks to the patient.

Therefore, it could be interesting for the future to introduce in the proposals of the robotic devices also functional activities and exercises aimed at the recovery of common activities in everyday life (Poli et al., 2013; World Health Organization, 2022).

## Limitations and future implications

The limitations that we have found in the conduct of this study are linked to a poor sensitivity of the scales administered, which have not allowed to make a more specific comparison with the indices obtained by the robot. It would be interesting to introduce a more complete evaluation of the fluidity and precision of the movement together with a functional evaluation of the upper limb in daily life activities. In fact, the evaluation of the ADL was carried out only with the administration of the BI, which is not specific enough to assess the impairment related to the functionality of the arm.

A second limitation concerns the low number of patients included and the absence of randomization. It would be interesting to evaluate the difference between a group that carries out the treatment in a facility with the possibility for the physiotherapist to intervene in the correction of the task, and the summation of the same therapy at home, with the assistance of the caregiver.

A third limit concerns the treatment time, which is shorter than the robotic home rehabilitation studies found in the literature. This time in fact varies between 6 and 12 weeks, while our study has provided a treatment time of only 2 weeks.

Finally, it was not possible to find in the literature a questionnaire validated in Italian that would consider items related to the use of the robot at home and that would evaluate both patients' and caregivers' acceptability. The questionnaire adopted in this study is not sufficiently structured to detect all the problematics that may occur during rehabilitation at home but was intended to assess in a simple and fast way the degree of acceptability of the patient and the caregiver toward the robot at home. It would be interesting to include a more structured questionnaire with more specific questions about the robot and compatibility in home use to assess whether there is a category of patients more suitable for this type of treatment.

In the future it would be interesting to include an economic feasibility study, considering the aspects related to the transport of the device, the need for internet connection, and aspects related to patient insurance at home.

## Conclusions

The presented study assessed the feasibility and the effectiveness of a home robotic rehabilitation program. Despite the small sample recruited, it was possible to record interesting and significant results, which support the use of robotic devices at home for the treatment of patients suffering from chronic stroke, even long after the acute event. These results are promising for this type of rehabilitation, so it would be interesting to continue the study on a larger sample, providing a longer therapy time and inserting a control group. The study presented is in fact a pilot study, without control group, so the results obtained should be considered as preliminary data and should be confirmed with better structured studies, such as Randomized Controlled Trials. It would also be useful to re-evaluate the patient to follow-up, to see if the results obtained are kept even at time from the use of the robot.

## Data availability statement

The original contributions presented in the study are included in the article/Supplementary material, further inquiries can be directed to the corresponding author.

## Ethics statement

The studies involving human participants were reviewed and approved by Campus Bio-Medico Ethical Committee. The patients/participants provided their written informed consent to participate in this study.

## Author contributions

LC, BC, FS, FF, MD'A, LR, and AP: data collection. FB, BC, LC, and MB: handwritten. GD, SM, VD, FC, and SS: revision. All authors contributed to the article and approved the submitted version.
