# Supplementary material for: Upper limb home-based robotic rehabilitation in chronic stroke patients: A pilot study
Source: Front Neurorobot. 2023 Mar 16;17:1130770. doi: 10.3389/fnbot.2023.1130770 (PMC10061073; doi:10.3389/fnbot.2023.1130770)
Supplement: Supplementary file 1 [file Data_Sheet_1.PDF]

QUESTIONARIO ACCETTABILITA' PROGETTO ARTES - ICON A DOMICILIO

Nome pz ______________________
Data somministrazione ______________________

Per il paziente:

Quanto ha gradito il trattamento a domicilio con il robot ICON?

0	1	2	3	4	5	6	7	8	9	10

Trova che questo tipo di trattamento a domicilio si concili con le sue attività quotidiane? SI | NO

Per il caregiver:

Il Suo carico assistenziale è aumentanto durante la terapia con ICON? SI | NO

Se ha risposto si alla domanda precedente, gentilmente quantifichi la sua risposta:

0	1	2	3	4	5	6	7	8	9	10

**Eventuali suggerimenti:**
