# Supplementary material for: Upper limb home-based robotic rehabilitation in chronic stroke patients: A pilot study
Source: Front Neurorobot. 2023 Mar 16;17:1130770. doi: 10.3389/fnbot.2023.1130770 (PMC10061073; doi:10.3389/fnbot.2023.1130770)
Supplement: Supplementary file 2 [file Data_Sheet_2.PDF]

ACCEPTABILITY QUESTIONNAIRE - HOME-BASED ICONE PROJECT

Patient Name_______________________

Date _______________________

For the patient:

How much did you enjoy the home treatment with the ICONE robot?

0	1	2	3	4	5	6	7	8	9	10

Do you find this type of home treatment compatible with your daily activities?

YES	NO

For the caregiver:

Is your care-load increasing during ICONE therapy?

YES	NO

If you answered yes to the previous question, please quantify your answer:

0	1	2	3	4	5	6	7	8	9	10

Any suggestions:
